# Disease Burden, Clinical Outcomes, and Quality of Life in People with Hemophilia A without Inhibitors in Europe: Analyses from CHESS II/CHESS PAEDs

**DOI:** 10.1055/s-0044-1785524

**Published:** 2024-04-15

**Authors:** Pratima Chowdary, Richard Ofori-Asenso, Francis Nissen, Enrico F. Grazzi, Martynas Aizenas, Katya Moreno, Tom Burke, Beatrice Nolan, Jamie O'Hara, Kate Khair

**Affiliations:** 1 Katharine Dormandy Haemophilia and Thrombosis Centre, Royal Free London NHS Foundation Trust, London, United Kingdom; 2 Real-World Data Enabling Platform, Roche Products Ltd, Welwyn Garden City, United Kingdom; 3 Department of Real-World Data, F. Hoffmann-La Roche Ltd, Basel, Switzerland; 4 Health Economics and Outcomes Research, HCD Economics, Daresbury, United Kingdom; 5 Department of Access Strategy, F. Hoffmann-La Roche Ltd, Basel, Switzerland; 6 Department of Product Development and Medical Affairs, F. Hoffmann-La Roche Ltd, Basel, Switzerland; 7 Department of Health and Social Care, University of Chester, Chester, United Kingdom; 8 Department of Haematology, Children's Health Ireland at Crumlin, Dublin, Ireland; 9 Department of Research, Haemnet, London, United Kingdom

**Keywords:** congenital hemophilia A, real-world data, disease burden

## Abstract

**Introduction** Limited data relating to treatment burden, quality of life, and mental health burden of hemophilia A (HA) are currently available.

**Aim** To provide a comprehensive overview of unmet needs in people with HA (PwHA) using data generated from the Cost of Haemophilia in Europe: a Socioeconomic Survey-II (CHESS II) and CHESS in the pediatric population (CHESS PAEDs) studies.

**Methods** CHESS II and CHESS PAEDs are cross-sectional surveys of European males with HA or hemophilia B (HB) aged ≥18 and ≤17 years, respectively. Participants with FVIII inhibitors, mild HA, or HB were excluded from this analysis, plus those aged 18 to 19 years. Annualized bleeding rates (ABRs), target joints, and other patient-reported outcomes were evaluated.

**Results** Overall, 468 and 691 PwHA with available data for the outcomes of interest were stratified by hemophilia severity and treatment regimen in CHESS II and CHESS PAEDs, respectively. In these studies, 173 (37.0%) and 468 (67.7%) participants received FVIII prophylaxis, respectively; no participants received the FVIII mimetic emicizumab or gene therapy. ABRs of 2.38 to 4.88 were reported across disease severity and treatment subgroups in both studies. Target joints were present in 35.7 and 16.6% of participants in CHESS II and CHESS PAEDS; 43.8 and 23.0% had problem joints. Chronic pain was reported by a large proportion of PwHA (73.9% in CHESS II; 58.8% in CHESS PAEDs). Participants also reported low EQ-5D scores (compared with people without HA), anxiety, depression, and negative impacts on their lifestyles due to HA.

**Conclusions** These analyses suggest significant physical, social, and mental burdens of HA, irrespective of disease severity. Optimization of prophylactic treatment could help reduce the burden of HA on patients.

## Introduction

Despite hemophilia A (HA) severity being classified by level of factor (F)VIII activity, the heterogeneity in bleeding phenotypes requires an individualized approach to treatment and prevention of bleeds for the best outcomes in individual patients. 1 Joint bleeding and resultant hemophilic arthropathy are common among people with HA (PwHA) of all ages and disease severities, despite the aim of prophylactic treatment to prevent recurring joint bleeds, demonstrating the necessity for improvement in the management of HA. 2 3 4 5 In the absence of prophylaxis, PwHA can experience frequent spontaneous bleeding and long-term health consequences, including premature mortality. 1

In previous analyses from the Cost of Haemophilia in Europe: a Socioeconomic Survey (CHESS) study, target joints were associated with, and a significant driver of, chronic pain, decreased health-related quality-of-life (HRQoL) scores, and increased direct costs in PwHA. 6 7

Treatment practice varies throughout Europe, with both prophylactic and on-demand treatment used in PwHA with moderate and severe disease. 8 Current treatment programs require optimization, as PwHA still present with high numbers of bleeds and target joints regardless of treatment approach. 8 In a separate analysis of people with moderate ( *n* = 864) and severe HA ( *n* = 1810), both groups showed similar annualized bleeding rates (ABRs) and hemophilia joint health scores, suggesting PwHA, particularly those with moderate HA, are not receiving optimal treatment. 9

Limited data relating to treatment burden via patient-reported outcomes in PwHA are available. 10 11 12 13 However, treatment burden, including time for preparation and administration of treatment, and infusion frequency, may impact treatment adherence. 14 In addition, many PwHA perceive multiple barriers to initiation of prophylaxis, resulting in reduced control of their hemophilia and thus limiting their daily activities. 11 13 15

Reports of the mental health burden of HA and its treatment are scarce, potentially underrepresenting the impact of HA in this population. 12 Previously published data highlight the impact of HA on quality of life (QoL) for both adults and children: PwHA experience anxiety and depression more often than the general population, which often goes undiagnosed and is associated with lower treatment adherence. 6 16 17 QoL data for the nonsevere population are also based on small datasets.

Further analysis of PwHA is required to accurately determine their unmet needs. This analysis provides a comprehensive overview of real-world outcomes in PwHA receiving replacement FVIII across Europe using data generated from the CHESS II and CHESS in the pediatric population (CHESS PAEDs) studies.

## Methods

### Study Design

We conducted retrospective analyses of the interim CHESS II and final CHESS PAEDs study populations, the methodologies of which have been reported previously. 3 18

CHESS II was a cross-sectional survey of adult men (aged ≥ 18 years) with HA or hemophilia B (HB) of any severity, with or without FVIII inhibitors, across eight European countries (Germany, Spain, France, Italy, Romania, the Netherlands, Denmark, and the United Kingdom), performed between 2019 and 2020. Participants voluntarily self-reported HRQoL and nondirect medical and indirect costs (relating to work productivity outcomes [patient/caregiver presenteeism/absenteeism or patient early retirement] and caregiver burden [hours of care per week]) via a paper-based questionnaire. Not all participants for whom clinical data are available completed a questionnaire, which explains variable participant numbers for some HRQoL endpoints. Health care providers completed online forms with clinical information from the participants' medical records and health care resource utilization, from which direct medical costs were derived.

CHESS PAEDs used a similar design with male children and adolescents (aged ≥1 years to ≤17 years of age with ≥12 months of data available) with moderate (FVIII levels 1–5%) or severe (FVIII level <1%) HA or HB, with or without inhibitors. Participants were sampled from five European countries (France, Germany, Italy, Spain, and the United Kingdom) between 2017 and 2018. Information on hemophilia-related costs and HRQoL was provided by either the participant or the caregiver (dependent on age and questionnaire).

For this analysis, data from all CHESS participants with inhibitors to FVIII, mild HA, or HB of any severity were excluded. CHESS PAEDs and CHESS II were conducted as separate studies, with a 2-year gap between data collection for the two studies. As a result of this, overlap in populations may be possible (i.e., patients who were 16–17 years old during CHESS PAEDs may have been 18–19 during data collection for CHESS II and therefore captured in both studies). Therefore, to avoid duplication, CHESS II participants aged 18 to 19 years were also excluded from this analysis. Participant treatment regimen was categorized as prophylaxis or “other” (on-demand or no treatment in the preceding 12 months). Only participants for whom data were available for the outcomes of interest are included in this analysis; no imputation was performed.

Physicians reported presence and frequency of recurrent bleeding into a joint (“target joints”) and joints with chronic pain and/or a limited range of movement (“problem joints”). Chronic pain was physician reported on a 4-level scale that included no chronic pain, and mild, moderate, or severe chronic pain; definitions are included in Supplementary Material S1 (available in the online version). Anxiety and depression were reported as comorbidities by the physicians, who abstracted the data from medical records. For participants in CHESS PAEDs, the age range for those with available anxiety and depression information was 4 to 17 years.

### Patient-Reported Health-Related Quality of Life

Patient-reported HRQoL was assessed using the EuroQol EQ-5D-5L questionnaire in CHESS II and the pediatric version of the EQ-5Q-3L (EQ-5D-Y) in CHESS PAEDs. The EQ-5D-5L comprises five domains (mobility, self-care, performance of usual activities, pain/discomfort, and anxiety/depression) with five levels. The EQ-5D-Y comprises the aforementioned five domains, with three levels. 19 CHESS PAEDs participants aged 8 to 17 completed the EQ-5D-Y themselves. CHESS II EQ-5D-5L index scores were derived via the United Kingdom value set for the EQ-5D-3L and cross-walk function. 20 EQ-5D-Y index scores were valued using the Spanish EQ-5D-Y value set. 21 Health state index utility scores were used to measure general health status and HRQoL, and were derived via algorithm from responses across all five domains; scores ranged from 0 (equivalent to “dead”) to 1 (“perfect health”). Derived scores of <0 (“worse than dead”) were possible. 22 Both CHESS study questionnaires used a 5-point Likert scale to evaluate hemophilia-related limitations related to reduction of social activities, exercise/physical activity, general opportunities, and frustration with the influence of hemophilia on their daily lives. Lower age limits were applied, when relevant, in QoL measures.

### Costs

Direct (associated with the health care system), indirect (not associated with the health care system), and total costs were estimated at the patient level for a 12-month retrospective period in accordance with the respective country-level unit cost databases.

Both the CHESS II and CHESS PAEDs studies received institutional review board approval, and informed consent, or assent where appropriate, was obtained.

## Results

### Study Population

The CHESS II analysis included 468 PwHA with a mean age of 38.7 years ( Tables 1 and 2 ). Of the 190/468 (40.6%) participants with moderate HA, 16 (8.4%) received primary FVIII prophylaxis (prophylaxis before the second joint bleed and aged <3 years); the majority (174 [91.6%]) received other treatment. Of the 278/468 (59.4%) participants with severe HA, 157 (56.5%) received FVIII prophylaxis ( *n* = 33 [11.9%] primary; *n* = 124 [44.6%] secondary [prophylaxis initiated after two or more joint bleeds, usually at age ≥3 years]) and 121 (43.5%) received other treatment. No participant received emicizumab or gene therapy.

**Table 1:** CHESS II demographics

Characteristic	( *N* = 468)
Age, y	
Mean (SD)	38.71 (13.95)
Median (IQR)	37.50 (22)
Age category, y, *n* (%)	
18–35	210 (44.9)
36–59	217 (46.4)
60+	41 (8.8)
Treatment approach, *n* (%)	
Prophylaxis	173 (37.0)
EHL FVIII	25 (14.5)
SHL or plasma-derived FVIII	131 (75.7)
Unknown	17 (9.8)
Other	295 (63.0)
EHL FVIII	27 (9.2)
SHL or plasma-derived FVIII	152 (51.5)
No FVIII administered in prior 12 mo a	115 (39.0)
Unknown	1 (0.3)
Severity, *n* (%)	
Moderate	190 (40.6)
Severe	278 (59.4)
Years since first diagnosis	
Mean (SD)	24.31 (15.24)
Median (IQR)	23 (20)
Years since first diagnosis category, *n* (%)	
< 5	66 (14.1)
5–10	30 (6.4)
> 10	372 (79.5)
Country, *n* (%)	
Germany	37 (7.9)
Spain	149 (31.8)
France	42 (9.0)
Italy	188 (40.2)
Romania	3 (0.6)
Holland	1 (0.2)
UK	48 (10.3)

Abbreviations: EHL, extended half-life; IQR, interquartile range; SD, standard deviation; SHL, standard half-life.

a All participants who had not received FVIII in the prior 12 months had moderate hemophilia A.

**Table 2:** CHESS II demographics by disease severity and treatment regimen

	Severity				Total ( *N* = 468)
	Moderate ( *n* = 190)		Severe ( *n* = 278)		
	Prophylaxis ( *n* = 16)	Other ( *n* = 174)	Prophylaxis ( *n* = 157)	Other ( *n* = 121)	
Age					
Mean (SD)	42.4 (16.9)	39.6 (14.6)	38.7 (14.0)	37.0 (12.4)	38.7 (13.9)
Median (IQR)	45.5 (29.5)	38 (21)	38 (22)	36 (17)	37.5 (22)
Age category, *n* (%)					
18–35	7 (43.8)	77 (44.3)	67 (42.7)	59 (48.8)	210 (44.9)
36–59	7 (43.8)	77 (44.3)	78 (49.7)	55 (45.5)	217 (46.4)
60+	2 (12.5)	20 (11.5)	12 (7.6)	7 (5.8)	41 (8.8)
Total	16 (100)	174 (100)	157 (100)	121 (100)	468 (100)
Treatment type, *n* (%)					
EHL FVIII	1 (6.3)	9 (5.2)	24 (15.3)	18 (14.9)	52 (11.1)
SHL or plasma-derived FVIII	2 (12.5)	50 (28.7)	129 (82.2)	102 (84.3)	283 (60.5)
No FVIII administered in prior 12 mo a	0 (0)	115 (66.1)	0 (0)	0 (0)	115 (24.6)
Unknown	13 (81.3)	0 (0)	4 (2.5)	1 (0.8)	18 (3.8)
Country					
Germany	0 (0.0)	16 (9.2)	14 (8.9)	7 (5.8)	37 (7.9)
Spain	11 (68.8)	53 (30.5)	47 (29.9)	38 (31.4)	149 (31.8)
France	0 (0.0)	16 (9.2)	13 (8.3)	13 (10.7)	42 (9.0)
Italy	5 (31.3)	68 (39.1)	67 (42.7)	48 (39.7)	188 (40.2)
Romania	0 (0.0)	2 (1.2)	0 (0.0)	1 (0.8)	3 (0.6)
Holland	0 (0.0)	1 (0.6)	0 (0.0)	0 (0.0)	1 (0.2)
UK	0 (0.0)	18 (10.3)	16 (10.2)	14 (11.6)	48 (10.3)

Abbreviations: EHL, extended half-life; IQR, interquartile range; SD, standard deviation; SHL, standard half-life.

a All participants who had not received FVIII in the prior 12 months had moderate hemophilia A.

The CHESS PAEDs analysis included 691 children and adolescents with HA, with a mean age of 10.3 years, of whom 53.8% ( *n* = 372) were aged <12 years ( Tables 3 and 4 ). Of 282/691 (40.8%) participants with moderate HA, 136 (48.2%) received FVIII prophylaxis and 146 (51.8%) received other treatment. Of 409/691 (59.2%) with severe HA, 332 (81.2%) received FVIII prophylaxis and 77 (18.8%) received other treatment. No participant received emicizumab or gene therapy.

**Table 3:** CHESS PAEDs demographics

Characteristic	*N* = 691
Age, y, mean (SD)	10.33 (4.70)
Age category, y, *n* (%)	
< 2	20 (2.9)
2–11	352 (50.9)
12–17	319 (46.2)
Treatment approach, *n* (%)	
Prophylaxis	468 (67.7)
EHL FVIII	81 (17.3)
SHL or plasma-derived FVIII	383 (81.8)
Unknown	4 (0.9)
Other	223 (32.3)
EHL FVIII	27 (12.1)
SHL or plasma-derived FVIII	109 (48.9)
No FVIII administered in prior 12 mo a	83 (37.2)
Unknown	4 (1.8)
Severity, *n* (%)	
Moderate	287 (40.8)
Severe	416 (59.2)
Country, *n* (%)	
France	148 (21.1)
Germany	112 (15.9)
Italy	167 (23.8)
Spain	168 (23.9)
UK	108 (15.4)

Abbreviations: EHL, extended half-life; SD, standard deviation; SHL, standard half-life.

a All participants who had not received FVIII in the prior 12 months had moderate hemophilia A.

**Table 4:** CHESS PAEDs demographics by disease severity and treatment regimen

	Severity				Total ( *N* = 691)
	Moderate ( *n* = 282)		Severe ( *n* = 409)		
	Prophylaxis ( *n* = 136)	Other ( *n* = 146)	Prophylaxis ( *n* = 332)	Other ( *n* = 77)	
Age					
Mean (SD)	10.4 (4.6)	11.0 (4.7)	10.3 (4.7)	9.1 (4.6)	10.3 (4.7)
Median (IQR)	10 (7)	12 (8)	11 (8)	10 (8)	10 (8)
Age category, *n* (%)					
< 2	5 (3.7)	5 (3.4)	8 (2.4)	2 (2.6)	20 (2.9)
2–11	67 (49.3)	67 (45.9)	169 (50.9)	49 (63.6)	352 (50.9)
12–17	64 (47.1)	74 (50.7)	155 (46.7)	26 (33.8)	319 (46.2)
Treatment type, *n* (%)					
EHL FVIII	21 (15.4)	9 (6.2)	60 (18.1)	18 (23.4)	108 (15.6)
SHL or plasma-derived FVIII	113 (83.1)0 (0)	54 (37.0)	270 (81.3)	55 (71.4)	492 (71.2)
No FVIII administered in prior 12 mo a	2 (1.5)	82 (56.2)	0 (0)	1 (0.1)	83 (12.0)
Unknown	0 (0)	1 (0.6)	2 (0.6)	3 (3.9)	8 (1.2)
Country, *n* (%)					
France	16 (11.8)	6 (4.1)	108 (32.5)	17 (22.0)	147 (21.3)
Germany	30 (22.1)	14 (9.6)	50 (15.1)	18 (23.4)	112 (16.2)
Italy	18 (13.2)	63 (43.2)	72 (21.7)	13 (16.9)	166 (24.0)
Spain	56 (41.2)	45 (30.8)	55 (16.6)	5 (6.5)	161 (23.3)
UK	16 (11.8)	18 (12.3)	47 (14.2)	24 (31.2)	105 (15.2)

Abbreviations: EHL, extended half-life; IQR, interquartile range; SD, standard deviation; SHL, standard half-life.

a All participants who had not received FVIII in the prior 12 months had moderate hemophilia A.

### Annualized Bleeding Rate

The mean ABR was 3.36 for the overall population of CHESS II ( Table 5 ) and 3.62 for the 691 PwHA with recorded ABRs in CHESS PAEDs ( Table 6 ).

**Table 5:** CHESS II outcomes evaluated by disease severity and treatment approach

	Severity				Total ( *N* = 468)
	Moderate ( *n* = 190)		Severe ( *n* = 278)		
	Prophylaxis ( *n* = 16)	Other ( *n* = 174)	Prophylaxis ( *n* = 157)	Other ( *n* = 121)	
ABR					
* n*	16	174	148	120	468
Mean (SD)	2.38 (1.82)	2.54 (2.97)	3.84 (3.09)	4.02 (9.18)	3.36 (5.33)
Median (IQR)	2.0 (2.0)	2.0 (2.0)	3.0 (3.5)	3.0 (3.0)	2.0 (3.0)
ABR category, *n* (%)					
0	1 (6.3)	27 (15.5)	12 (7.6)	9 (7.4)	49 (10.5)
1	4 (25.0)	53 (30.5)	25 (15.9)	24 (19.8)	105 (22.4)
2+	11 (68.8)	94 (54.0)	120 (76.4)	88 (72.7)	314 (67.1)
Target joint number category, *n* (%)					
None	10 (62.5)	135 (77.6)	90 (57.3)	66 (54.5)	301 (64.3)
1 target joint	5 (31.3)	22 (12.6)	36 (22.9)	28 (23.1)	91 (19.4)
2+ target joints	1 (6.3)	17 (9.8)	31 (19.7)	27 (22.3)	76 (16.2)
Problem joint number category, *n* (%)					
None	4 (25.0)	112 (64.4)	79 (50.3)	68 (56.2)	263 (56.2)
1 problem joint	9 (56.3)	35 (20.1)	41 (26.1)	32 (16.4)	117 (25.0)
2+ problem joints	3 (18.7)	27 (15.5)	37 (23.6)	21 (17.4)	88 (18.8)
Current chronic pain level, *n* (%)					
No pain	3 (18.8)	59 (33.9)	33 (21.0)	27 (22.3)	122 (26.1)
Mild pain	5 (31.3)	81 (46.6)	60 (38.2)	43 (35.5)	189 (40.4)
Moderate pain	8 (50.0)	31 (17.8)	49 (31.2)	38 (31.4)	126 (26.9)
Severe pain	0 (0.0)	3 (1.7)	15 (9.6)	13 (10.7)	31 (6.6)
Participants with anxiety, *n* (%)	8 (50.0)	28 (16.1)	34 (21.7)	16 (13.2)	86 (18.4)
Participants with depression, *n* (%)	3 (18.8)	20 (11.5)	12 (7.6)	14 (11.6)	49 (10.5)

Abbreviations: ABR, annualized bleeding rate; IQR, interquartile range; SD, standard deviation.

**Table 6:** CHESS PAEDs outcomes evaluated by disease severity and treatment approach

	Severity				Total ( *N* = 691)
	Moderate ( *n* = 282)		Severe ( *n* = 409)		
	Prophylaxis ( *n* = 136)	Other ( *n* = 146)	Prophylaxis ( *n* = 332)	Other ( *n* = 77)	
ABR					
Mean (SD)	4.01 (9.73)	4.32 (9.86)	2.86 (4.93)	4.88 (16.15)	3.62 (8.94)
Median (IQR)	1.0 (3.5)	2.0 (5.0)	2.0 (3.0)	2.0 (3.0)	2 (4)
ABR category, *n* (%)					
0	43 (31.6)	38 (26.0)	85 (25.6)	17 (22.1)	183 (26.5)
1	26 (19.1)	33 (22.6)	68 (20.5)	7 (9.0)	134 (19.4)
2+	67 (49.3)	75 (51.4)	179 (53.9)	53 (68.8)	374 (54.1)
Target joint number category, *n* (%)					
None	112 (82.4)	139 (95.2)	260 (78.3)	65 (84.4)	576 (83.4)
1 target joint	18 (13.2)	6 (4.1)	61 (18.4)	10 (13.0)	95 (13.7)
2+ target joints	6 (4.4)	1 (0.7)	11 (3.3)	2 (2.6)	20 (2.9)
Problem joint number category, *n* (%)					
None	101 (74.3)	134 (91.8)	233 (70.2)	64 (83.1)	532 (77.0)
1 problem joint	22 (16.2)	8 (5.5)	78 (23.5)	10 (13.0)	118 (17.1)
2+ problem joints	13 (5.9)	4 (2.7)	21 (6.3)	3 (3.9)	41 (5.9)
Current chronic pain level, *n* (%)					
No pain	58 (42.6)	74 (50.7)	126 (38.0)	27 (35.1)	285 (41.2)
Mild pain	52 (38.2)	64 (43.8)	147 (44.3)	33 (42.9)	296 (42.8)
Moderate pain	26 (19.1)	8 (5.5)	57 (17.2)	17 (22.1)	108 (15.6)
Severe pain	0 (0)	0 (0)	2 (0.6)	0 (0)	2 (0.3)
Participants with anxiety, *n* (%)	10 (7.4)	13 (8.9)	26 (7.8)	7 (9.1)	56 (8.1)
Participants with depression, *n* (%)	3 (2.2)	5 (3.4)	4 (1.2)	1 (1.3)	13 (1.9)

Abbreviations: ABR, annualized bleeding rate; IQR, interquartile range; SD, standard deviation.

Mean ABRs were similar between treatment regimens in the moderate population: 2.38 in those on prophylaxis ( *n* = 16) and 2.54 in participants on other treatment ( *n* = 174) in CHESS II. In CHESS PAEDs, the mean ABR was 4.01 in participants on prophylaxis ( *n* = 136) and 4.32 in those on other treatment ( *n* = 146). For participants with severe HA, mean ABRs in CHESS II were 3.84 in those receiving prophylaxis ( *n* = 157) and 4.02 in those receiving other treatment ( *n* = 121). In CHESS PAEDs, the mean ABR was 2.86 on prophylaxis ( *n* = 332) and 4.88 on other treatment ( *n* = 77).

### Target Joints

In CHESS II, 167 participants (35.7%) had ≥1 target joint ( Table 5 ); in CHESS PAEDs, 115 participants had ≥1 target joint (16.6%; Table 6 ).

The proportion of participants with target joints was numerically similar across disease severities and treatment regimens in both studies. In the moderate population of CHESS II, 37.5 and 22.4% of participants receiving prophylaxis and other treatment, respectively, had target joints; in the severe population, these percentages were 42.7 and 45.5%. In CHESS PAEDs, target joints were present in 17.6% of participants with moderate HA receiving prophylaxis and 4.8% receiving other treatment, and in 21.7% of participants with severe HA receiving prophylaxis and 15.6% receiving other treatment.

### Problem Joints

In CHESS II, 205 participants (43.8%) had ≥1 problem joint ( Table 5 ); in CHESS PAEDs, 159 participants had ≥1 problem joint (23.0%; Table 6 ).

In CHESS II, more participants with severe disease reported ≥1 problem joint (47.1%) compared with the moderate population (38.9%). Between disease severity populations, similar proportions of participants reported 1 problem joint; however, a greater proportion in the severe population reported ≥2 problem joints. In CHESS PAEDs, reports of problem joints were similar across disease severity and treatment regimen subgroups, although they were reported less frequently in participants receiving other treatment for moderate HA, compared with prophylaxis. Problem joints were reported in participants aged 1 to 17 years, although 77% of reports were from participants aged > 8 years.

### Chronic Pain

In CHESS II, 346 participants (73.9%) had chronic pain ( Table 5 ). In CHESS PAEDs, 406 participants (58.8%; aged 1–17) had chronic pain ( Table 6 ).

Across disease severities in CHESS II, a greater proportion of those with severe disease had moderate or severe pain compared with those who had moderate HA (31.3 vs. 20.5% for moderate pain; 10.1 vs. 1.6% for severe pain). However, similar proportions of participants had mild or no pain across disease severities and treatment regimens, with the exception of PwHA receiving other treatment for moderate disease, where a larger proportion reported no pain.

In CHESS PAEDs, similar proportions of participants reported mild or no pain irrespective of disease severity or treatment regimen. A greater proportion of the moderate population reported no pain than the severe population (46.8 vs. 37.4%). Only two participants, receiving prophylaxis for severe disease, reported severe pain.

### Anxiety and Depression

In CHESS II and CHESS PAEDS, 86 (18.4%) and 56 (8.1%; aged 3–17 years) participants, respectively, had feelings of anxiety ( Tables 5 and 6 ). Reports of anxiety in CHESS II were greater in those receiving prophylaxis across both disease severities: 50.0% of participants receiving prophylaxis versus 16.1% receiving other treatment in the moderate population and 21.7 versus 13.2% in the severe population.

Depression was reported in 49 (10.5%) participants in CHESS II ( Table 5 ), and 13 (1.9%; aged 6–17 years) participants in CHESS PAEDs ( Table 6 ). The proportions of participants with depression were consistent across disease severity and treatment regimen subgroups in both studies. Reports of depression were lower in all subgroups for the pediatric population compared with adults.

### Quality of Life

In CHESS II, the mean EQ-5D score was 0.71 for the overall population and was similar across disease severity and treatment regimen subgroups (0.66–0.76; Table 7 ).

**Table 7:** CHESS II quality-of-life measures evaluated by disease severity and treatment approach

	Severity				Total ( *N* = 468)
	Moderate ( *n* = 190)		Severe ( *n* = 278)		
	Prophylaxis ( *n* = 16)	Other ( *n* = 174)	Prophylaxis ( *n* = 157)	Other ( *n* = 121)	
EQ-5D					
* n*	5	65	81	54	205
Mean (SD)	0.66 (0.36)	0.76 (0.17)	0.68 (0.28)	0.69 (0.22)	0.71 (0.24)
Median (IQR)	0.77 (0.06)	0.74 (0.29)	0.71 (0.32)	0.71 (0.30)	0.71 (0.3)
Had to reduce or give up social activities, *n* (%)	*n* = 5	*n* = 65	*n* = 82	*n* = 53	*n* = 205
Strongly agree	1 (20.0)	4 (6.2)	21 (25.6)	15 (28.3)	41 (20.0)
Agree	3 (60.0)	19 (29.2)	25 (30.5)	22 (41.5)	69 (33.7)
Neither agree nor disagree	0 (0.0)	15 (23.1)	14 (17.1)	6 (11.3)	35 (17.1)
Disagree	1 (20.0)	16 (24.6)	14 (17.1)	7 (13.2)	38 (18.5)
Strongly disagree	0 (0.0)	11 (16.9)	8 (9.8)	3 (5.7)	22 (10.7)
Had to reduce or give up exercise, *n* (%)	*n* = 5	*n* = 65	*n* = 82	*n* = 53	*n* = 205
Strongly agree	3 (60.0)	9 (13.8)	26 (31.7)	15 (28.3)	53 (25.9)
Agree	1 (20.0)	17 (26.2)	31 (37.8)	19 (35.8)	68 (33.2)
Neither agree nor disagree	1 (20.0)	15 (23.1)	6 (7.3)	7 (13.2)	29 (14.1)
Disagree	0 (0.0)	14 (21.5)	11 (13.4)	11 (20.8)	36 (17.6)
Strongly disagree	0 (0.0)	10 (15.4)	8 (9.8)	1 (1.9)	19 (9.3)
Miss out on opportunities, *n* (%)	*n* = 5	*n* = 65	*n* = 82	*n* = 53	*n* = 205
Strongly agree	0 (0)	5 (7.7)	19 (23.2)	13 (24.5)	37 (18.0)
Agree	4 (80.0)	16 (24.6)	35 (42.7)	22 (41.5)	77 (37.6)
Neither agree nor disagree	0 (0.0)	18 (27.7)	10 (12.2)	5 (9.4)	33 (16.1)
Disagree	1 (20.0)	16 (24.6)	9 (11.0)	11 (20.8)	37 (18.0)
Strongly disagree	0 (0)	10 (15.4)	9 (11.0)	2 (3.8)	21 (10.2)
Feel frustrated by the influence on lifestyle, *n* (%)	*n* = 5	*n* = 65	*n* = 82	*n* = 53	*n* = 205
Strongly agree	1 (20.0)	4 (6.2)	20 (24.4)	10 (18.9)	35 (17.1)
Agree	2 (40.0)	17 (26.2)	29 (35.4)	22 (41.5)	70 (34.1)
Neither agree nor disagree	0 (0)	15 (23.1)	11 (13.4)	8 (15.1)	34 (16.6)
Disagree	1 (20.0)	18 (27.7)	12 (14.6)	12 (22.6)	43 (21.0)
Strongly disagree	1 (20.0)	11 (16.9)	10 (12.2)	1 (1.9)	23 (11.2)

Abbreviations: IQR, interquartile range; SD, standard deviation.

In CHESS PAEDs, the mean EQ-5D-Y score was 0.65 for the overall population (age range: 8–17 years) and ranged between 0.65 and 0.70 for those with moderate HA receiving prophylaxis or any treatment for severe disease ( Table 8 ). However, participants with moderate HA receiving other treatment reported a lower score of 0.50.

**Table 8:** CHESS PAEDs quality-of-life measures evaluated by disease severity and treatment approach

	Severity				Total ( *N* = 691)
	Moderate ( *n* = 282)		Severe ( *n* = 409)		
	Prophylaxis ( *n* = 136)	Other ( *n* = 146)	Prophylaxis ( *n* = 332)	Other ( *n* = 77)	
EQ-5D score for children > 8 y					
* n*	17	10	76	18	121
Mean (SD)	0.70 (0.23)	0.50 (0.53)	0.65 (0.25)	0.69 (0.17)	0.65 (0.27)
Median (IQR)	0.71 (0.27)	0.70 (0.66)	0.65 (0.29)	0.70 (0.22)	0.68 (0.27)
Had to reduce or give up social activities, *n* (%)	*n* = 28	*n* = 17	*n* = 100	*n* = 28	*n* = 173
Strongly agree	4 (14.3)	1 (5.9)	5 (5.0)	2 (7.1)	12 (6.9)
Agree	8 (28.6)	4 (23.5)	17 (17.0)	4 (14.3)	33 (19.1)
Neither agree nor disagree	5 (17.9)	2 (11.8)	21 (21.0)	7 (25.0)	35 (20.2)
Disagree	9 (32.1)	8 (47.1)	50 (50.0)	14 (50.0)	81 (46.8)
Strongly disagree	2 (7.1)	2 (11.8)	7 (7.0)	1 (3.6)	12 (6.9)
Had to reduce or give up exercise, *n* (%)	*n* = 28	*n* = 18	*n* = 100	*n* = 28	*n* = 174
Strongly agree	2 (7.1)	0 (0.0)	4 (4.0)	2 (7.1)	8 (4.6)
Agree	7 (25.0)	2 (11.1)	5 (5.0)	8 (28.6)	22 (12.6)
Neither agree nor disagree	5 (17.9)	7 (38.9)	29 (29.0)	7 (25.0)	48 (27.6)
Disagree	11 (39.3)	8 (44.4)	46 (46.0)	9 (32.1)	74 (42.5)
Strongly disagree	3 (10.7)	1 (5.6)	16 (16.0)	2 (7.1)	22 (12.6)
Miss out on opportunities, *n* (%)	*n* = 28	*n* = 18	*n* = 100	*n* = 28	*n* = 174
Strongly agree	2 (7.1)	0 (0)	3 (3.0)	2 (7.1)	7 (4.0)
Agree	5 (17.9)	5 (27.8)	10 (10.0)	6 (21.4)	26 (14.9)
Neither agree nor disagree	12 (42.9)	4 (22.2)	32 (32.0)	11 (39.3)	59 (33.9)
Disagree	7 (25.0)	8 (44.4)	49 (49.0)	9 (32.1)	73 (42.0)
Strongly disagree	2 (7.1)	1 (5.6)	6 (6.0)	0 (0)	9 (5.2)
Feel frustrated by the influence on lifestyle, *n* (%)	*n* = 28	*n* = 18	*n* = 100	*n* = 28	*n* = 174
Strongly agree	2 (7.1)	0 (0)	2 (2.0)	0 (0)	4 (2.3)
Agree	5 (17.9)	4 (22.2)	12 (12.0)	4 (14.3)	25 (14.4)
Neither agree nor disagree	11 (39.3)	8 (44.4)	30 (30.0)	11 (39.3)	60 (34.5)
Disagree	8 (28.6)	5 (27.8)	48 (48.0)	13 (46.4)	74 (42.5)
Strongly disagree	2 (7.1)	1 (5.6)	8 (8.0)	0 (0)	11 (6.3)
Parent/guardian providing care for a pediatric relative with hemophilia, *n* (%)	*n* = 30	*n* = 18	*n* = 97	*n* = 26	*n* = 171
Yes	18 (60.0)	12 (66.7)	77 (79.4)	13 (50.0)	120 (70.2)
No	12 (40.0)	6 (33.3)	20 (20.6)	13 (50.0)	51 (29.8)
Hours spent caring in a week					
* n*	17	11	77	13	118
Mean (SD)	24.88 (20.92)	40.45 (34.08)	19.70 (24.61)	12.00 (10.26)	21.54 (24.72)
Median (IQR)	15.0 (20.0)	40.0 (71.0)	12.0 (16.0)	8.0 (4.0)	12 (18)
Caregiver duties prevent you from working/working more hours, *n* (%)	*n* = 12	*n* = 10	*n* = 60	*n* = 11	*n* = 93
Yes	1 (5.6)	3 (25.0)	9 (11.7)	7 (53.8)	20 (21.5)
No	6 (50.0)	6 (60.0)	44 (73.3)	4 (36.4)	60 (64.5)
Don't know	5 (41.7)	1 (10.0)	7 (11.7)	0 (0)	13 (14.0)

Abbreviations: IQR, interquartile range; SD, standard deviation.

In both CHESS II and CHESS PAEDs, participants reported they had to reduce or give up social activities or exercise, missed out on opportunities, and felt frustrated by the influence of HA on their lifestyle ( Tables 7 and 8 ).

ABR, target and problem joints, chronic pain, anxiety and depression, and QoL outcomes split by country of origin for CHESS II and CHESS PAEDs are presented in Supplementary Tables S1 and S2 (available in the online version), respectively.

### Costs

Direct, indirect, and total costs are summarized in Table 9 for CHESS II and Table 10 for CHESS PAEDs. In both studies, direct medical costs and total costs were higher in participants with severe HA versus participants with moderate HA.

**Table 9:** CHESS II direct, indirect, and total costs

	Severity				Total ( *N* = 468)
	Moderate ( *n* = 190)		Severe ( *n* = 278)		
	Prophylaxis ( *n* = 16)	Other ( *n* = 174)	Prophylaxis ( *n* = 157)	Other ( *n* = 121)	
Direct costs, EUR					
* n*	16	174	157	121	468
Mean (SD)	7,920 (7,245)	20,005 (47,023)	266,568 (204,870)	89,893 (143,376)	120,376 (178,147)
Median (IQR)	4,139 (8,746)	4,112 (13,279)	226,910 (313,460)	36,408 (68,481)	28,575 (155,660)
Indirect costs (PPIE only), EUR					
* n*	5	65	82	54	206
Mean (SD)	14,274 (14,219)	3,283 (9,934)	8,539 (13,862)	9,181 (14,769)	7,188 (13,225)
Median (IQR)	14,536 (27,689)	0 (425)	1,522 (9,691)	1,087 (11,995)	0 (69,223)
Total costs, EUR * n*	5	65	82	54	206
Mean (SD)	21,039 (18,840)	23,711 (52,318)	295,630 (216,231)	117,147 (188,619)	156,379 (206,695)
Median (IQR)	21,316 (34,724)	3,941 (18,128)	254,866 (311,266)	54,455 (62,799)	62,520 (232,413)

Abbreviations: EUR, Euros; IQR, interquartile range; PPIE, patient and public involvement and engagement; SD, standard deviation.

**Table 10:** CHESS PAEDs direct, indirect, and total costs

	Severity				Total ( *N* = 703)
	Moderate ( *n* = 287)		Severe ( *n* = 416)		
	Prophylaxis ( *n* = 141)	Other ( *n* = 146)	Prophylaxis ( *n* = 335)	Other ( *n* = 81)	
Direct medical costs, EUR					
* n*	141	146	335	81	703
Mean (SD)	75,466 (106,165)	9,257 (21,222)	113,373 (112,214)	15,847 (34,233)	72,910 (102,305)
Median (IQR)	35,893 (88,540)	4,378 (6,282)	77,780 (123,471)	10,114 (11,010)	28,664 (96,302)
Indirect costs (PPIE only), EUR					
* n*	30	18	100	28	176
Mean (SD)	465 (2,546)	3,499 (98,07)	887 (3,627)	2,509 (7,174)	1,340 (51,723)
Median (IQR)	0 (0)	0 (0)	0 (0)	0 (697)	0 (0)
Total costs, EUR					
* n*	30	18	100	28	176
Mean (SD)	79,389 (90,513)	23,897 (29,059)	99,634 (97,826)	32,206 (56,255)	77,710 (90,808)
Median (IQR)	48,437 (48,722)	13,482 (33,726)	70,551 (120,394)	17,228 (17,148)	44,701 (87,515)

Abbreviations: EUR, Euros; IQR, interquartile range; PPIE, patient and public involvement and engagement; SD, standard deviation.

## Discussion

This retrospective analysis of the CHESS II and CHESS PAEDs population studies reveals high ABRs and prevalence of target joints across disease severities and treatment regimens in PwHA. Such characteristics may contribute to chronic pain and reduced QoL measures, leading to physical, mental, social, and economic impacts in adults, children, and their caregivers. These findings suggest potential suboptimal administration of HA treatment, including prophylaxis, in PwHA in Europe.

Despite the larger proportion of people with severe HA compared with moderate HA in this analysis, a smaller proportion of adults in CHESS II received FVIII prophylactic treatment than other treatment. This demonstrates a considerable gap between the current standards of care and the aspirations laid out in the recent World Federation of Hemophilia (WFH) guidelines. 1

Surprisingly, in CHESS II, ABRs and numbers of target joints were similar regardless of treatment regimen, perhaps reflecting the implementation of prophylaxis in patients who are considered to have severe bleeding phenotype. In CHESS PAEDs, target joints were present in similar proportions across subgroups; however, ABR values were higher in participants receiving other treatment versus prophylaxis, especially in the severe population, in line with clinical experience. These results indicate suboptimal administration of prophylactic treatment across PwHA in Europe, as optimal treatment aims to decrease ABRs and reduce numbers of target joints. 1 The failure to achieve the latter goal particularly reflects the recurrence of joint bleeds and inadequate prophylaxis.

Suboptimal outcomes may also indicate barriers to prophylaxis uptake in children/adolescents, such as parental inability to administer treatment, fear of needles, considerable burden of frequent injections, or not wanting to stand out from their peers. Additionally, ABRs and target joint scores for moderate and severe populations were similar, suggesting that many people with moderate HA may have inadequate bleed control. This issue has also been identified previously. 17

High ABRs and the presence of target joints in PwHA are associated with compromised joint integrity, such as chronic synovitis or hemophilic arthropathy, leading to the development of problem joints, generally characterized by chronic pain and/or a limited range of movement. In these analyses, problem joints were more common in the adult population compared with children and were more prevalent in the severe population compared with other participants in CHESS II, representing cumulative joint damage over the lifetime of the patient. A previous analysis of problem joints in CHESS II and CHESS PAEDs indicated an association between problem joints and worsening clinical and QoL outcomes across disease severities. 23 Indeed, higher levels of chronic pain were reported in CHESS II than CHESS PAEDs. However, participants in both analyses had similar prevalence of chronic pain, regardless of treatment, indicating a need for more effective pain management in PwHA. 7

The prevalence of depression and anxiety were similar across disease severity groups in both adult and pediatric populations, although they were numerically higher in those with moderate versus severe disease, which may warrant further examination of the factors impacting mental health in these groups. Subgroup analyses demonstrated a higher prevalence of anxiety in adults receiving prophylaxis than in those receiving other treatment, perhaps reflecting the burden perceived by participants; however, this was in a comparatively smaller subgroup. Depression was reported least frequently in adults with severe HA receiving prophylaxis and most frequently in those receiving prophylaxis for moderate HA. This may be related to the delayed initiation of prophylaxis in those with moderate HA, as poor disease outcomes manifest later in the course of disease. Furthermore, a participant may be frustrated by the treatment burden of prophylaxis should they not see equivalent improvements in their disease burden. As a result, increased monitoring, the use of a validated self-assessment tool, and the implementation of a mental health professional in the multidisciplinary team would all be valuable steps toward tackling mental health challenges in people with hemophilia. Indeed, WFH guidelines recognize the importance of psychosocial care in the management of hemophilia; recommendations are provided on how the hemophilia treatment center social worker and/or other members of the care team can support people with hemophilia and their caregivers in challenges with mental health, such as anxiety or depression. 1

The prevalence of anxiety observed in adults in this analysis was slightly lower than in previous studies of PwHA. 17 24 In an analysis of adults with hemophilia, the presence of depression, anxiety, and pain were found to be interrelated. 7 It is therefore possible that reducing the depression and anxiety identified in these populations may be achieved via improvements to pain management.

EQ-5D scores reported in CHESS II were low compared with the norms for the general population published previously. 25 26 27 Similar scores were reported in CHESS PAEDs, indicating a similar deficiency in HRQoL, although general population norms are unavailable for comparison. Those receiving prophylaxis reported greater impact on their QoL compared with those receiving other treatment. However, the majority of those receiving prophylaxis are likely to have severe HA, per the treatment guidelines, and results may therefore be impacted primarily by disease severity, rather than treatment received. 1

Across both study populations, both PwHA and caregivers reported impacts on their daily lives, including missed opportunities, reduced social activities, and frustration with the influence of hemophilia over their lives. Lack of social support from family and friends has previously been linked to the presence of depressive symptoms in PwHA, demonstrating the potential to improve mental welfare by improving symptoms that affect daily activities and hence allow PwHA to engage in social activities. 7 28

Such impacts on the lives of PwHA may be consequences of the disease burden of HA. Current disease characteristics of PwHA in Europe highlight a remaining unmet need for improvements in the standard of care, the achievement of which could reduce the burden of HA and therefore tackle subsequent impacts on QoL and daily activities. To this point, there is growing evidence suggesting subcutaneously administered emicizumab, which has been characterized as a less burdensome prophylaxis option than standard-of-care FVIII replacement, can provide improved HRQoL. 29 Future analyses including therapies such as emicizumab, gene therapy, and other novel treatment options could provide necessary insight into whether the changing treatment landscape translates into improvements in the suboptimal outcomes reported here in participants receiving FVIII replacement.

## Limitations

The results of these analyses should be interpreted in the context of limitations inherent to cross-sectional studies measuring outcomes from defined snapshots in time. A further limitation may be the sample size, specifically in the number of participants with moderate HA receiving prophylaxis; therefore, interpretation of the results should take this into consideration. Participation in the CHESS studies was entirely voluntary and contingent on patients visiting their physician, so selection bias cannot be excluded. Additionally, there may be a degree of recall bias in patient-reported outcomes surveys and possible medical chart data abstraction errors. Possible country-specific differences in care provision and health care systems should also be taken into consideration when interpreting these results.

## Conclusion

These analyses highlight the unmet physical, mental, social, and economic needs faced by PwHA in Europe. Almost half of all PwHA, regardless of age or disease severity, still experienced ABRs ≥2, and target joints and problem joints were prevalent across all subgroups. Such outcomes highlight the suboptimal administration of prophylactic treatment in PwHA in Europe. Disease burden was associated with chronic pain, anxiety and depression, and negative impacts on QoL regardless of disease severity. These observations may reflect the need for improved prophylactic treatment options and optimal administration of such treatments. Our findings may be useful for defining new treatment strategies that decrease not only the physical burden, but also the social and mental burden of hemophilia for people in Europe.

## Notes

**What is known about this topic?**

- Despite the availability of improved treatment options for people with hemophilia A (HA) in Europe, joint bleeding and resultant hemophilic arthropathy are common among people with HA of all ages.
- As a result, there are unmet needs among adults and children with moderate or severe HA in Europe.

**What does this paper add?**

- This analysis highlights the suboptimal administration of prophylactic treatment in adults and children with moderate or severe HA in Europe.
- There also appears to be association of disease burden with subsequent impacts on key patient-reported outcomes, such as prevalence of chronic pain, depression, and anxiety, regardless of disease severity.
- Improved prophylactic treatment options and optimal administration of these treatments may facilitate new treatment strategies that decrease the burden of HA in Europe, and bring us closer to achieving the goals laid out in the latest WFH guidelines.
