# Supplementary material for: Disease Burden, Clinical Outcomes, and Quality of Life in People with Hemophilia A without Inhibitors in Europe: Analyses from CHESS II/CHESS PAEDs
Source: TH Open. 2024 Apr 15;8(2):e181–93. doi: 10.1055/s-0044-1785524 (PMC11018388; doi:10.1055/s-0044-1785524)
Supplement: Supplementary file 1 — Supplementary Material [file 10-1055-s-0044-1785524-s23110046.pdf]

Supplementary Material S1 Glossary

Term	Definition
Hemophilia A	A deficiency or absence of factor VIII. The most common form of hemophilia, also called "classic hemophilia"
Moderate hemophilia	A factor VIII or IX level ranging from 1 to 5% of normal blood levels
Severe hemophilia	A factor VIII or IX level below 1% of normal blood levels
Prophylaxis	A treatment regimen to prevent bleeds
Target joint	A joint that has had repeated bleeds, or at least four bleeds within a 6-mo period
Problem joint	A joint with chronic pain and/or a limited range of movement
Treatment adherence	The process by which patients take their medication as prescribed
No chronic pain	No functional deficit; no analgesic use (except with acute hemarthrosis)
Mild chronic pain	Pain that does not interfere with occupation nor with activities of daily living (ADL) but may require occasional non-narcotic analgesic
Moderate chronic pain	Pain causing partial or occasional interference with occupation or ADL; the individual makes use of non-narcotic medications
Severe chronic pain	Pain interfering with occupation or ADL; requires frequent use of non-narcotic and narcotic medications

Supplementary Table S1 CHESS II outcomes evaluated by country of origin

	Country							
	Germany	Spain	France	Italy	Romania	Holland	UK	Total
	n = 37	*n* = 149	*n* = 42	*n* = 188	*n* = 3	*n* = 1	*n* = 48	*N* = 468
ABR								
n	37	149	42	188	3	1	48	468
Mean (SD)	1.59 (1.55)	3.84 (8.55)	2.24 (2.23)	3.49 (3.07)	0.67 (1.15)	1.00 (N/A)	4.00 (1.69)	3.35 (5.39)
Median (IQR)	1 (2)	2 (3)	2 (2)	3 (3)	0 (2)	1 (0)	4 (1.5)	2 (3)
ABR category, *n* (%)								
0	10 (27.0)	14 (9.4)	7 (16.7)	16 (8.5)	2 (66.7)	0 (0.0)	0 (0.0)	49 (10.5)
1	12 (32.4)	42 (28.2)	13 (31.0)	35 (18.6)	0 (0.0)	1 (100.0)	2 (4.2)	105 (22.4)
2+	15 (40.5)	93 (62.4)	22 (52.4)	137 (72.9)	1 (33.3)	0 (0.0)	46 (95.8)	314 (67.1)
Number of target joints								
n	37	149	42	188	3	1	48	468
Mean (SD)	0.32 (0.67)	0.82 (1.15)	0.45 (0.94)	0.59 (0.83)	0.00 (0.00)	0.00 (0.0)	0.17 (0.48)	0.58 (0.93)
Median (IQR)	0 (0)	0 (1)	0 (0)	0 (1)	0 (0)	0 (0)	0 (0)	0 (1)
Target joint number category, *n* (%)								
None	28 (75.7)	82 (55.0)	32 (76.2)	113 (60.1)	3 (100.0)	1 (100.0)	42 (87.5)	301 (64.3)
1 target joint	7 (18.9)	32 (21.5)	4 (9.5)	44 (23.4)	0 (0.0)	0 (0.0)	4 (8.3)	91 (19.4)
2+ target joints	2 (5.4)	35 (23.5)	6 (14.3)	31 (16.5)	0 (0.0)	0 (0.0)	2 (4.2)	76 (16.2)
Current chronic pain level, *n* (%)								
No pain	12 (32.4)	48 (32.2)	23 (54.8)	32 (17.0)	1 (33.3)	0 (0.0)	6 (12.5)	122 (26.1)
Mild pain	16 (43.2)	48 (32.2)	18 (42.9)	89 (47.3)	1 (33.3)	1 (100.0)	16 (33.3)	189 (40.4)
Moderate pain	9 (24.3)	44 (29.5)	1 (2.4)	53 (28.2)	0 (0.0)	0 (0.0)	19 (39.6)	126 (26.9)
Severe pain	0 (0.0)	9 (6.0)	0 (0.0)	14 (7.4)	1 (33.3)	0 (0.0)	7 (14.6)	31 (6.6)
% with anxiety, *n* (%)	2 (5.4)	36 (24.2)	3 (7.1)	39 (20.7)	0 (0.0)	0 (0.0)	6 (12.5)	86 (18.4)
% with depression, *n* (%)	2 (5.4)	20 (13.4)	2 (4.8)	22 (11.7)	0 (0.0)	0 (0.0)	3 (6.3)	49 (10.5)

 © 2024. The Author(s).

Supplementary Table S1 (*Continued*)

	Country							
	Germany	Spain	France	Italy	Romania	Holland	UK	Total
	n = 37	*n* = 149	*n* = 42	*n* = 188	*n* = 3	*n* = 1	*n* = 48	*N* = 468
EQ-5D								
n	4	80	26	83	No	No	12	205
Mean (SD)	0.79 (0.12)	0.72 (0.20)	0.85 (0.15)	0.64 (0.28)	observations	observations	0.75 (0.18)	0.71 (0.3)
Median (IQR)	0.8 (0.2)	0.74 (0.29)	0.88 (0.27)	0.67 (0.31)			0.68 (0.36)	0.71 (0.24)
Had to reduce or give up social activities, *n* (%)	*n* = 4	*n* = 80	*n* = 25	*n* = 84	*n* = 0	*n* = 0	*n* = 12	*n* = 205
Strongly agree	0 (0.0)	18 (22.5)	6 (24.0)	17 (20.2)	0 (0.0)	0 (0.0)	0 (0.0)	41 (20.0)
Agree	1 (25.0)	29 (36.3)	5 (20.0)	30 (35.7)	0 (0.0)	0 (0.0)	4 (33.3)	69 (33.7)
Neither agree nor disagree	0 (0.0)	14 (17.5)	8 (32.0)	13 (15.5)	0 (0.0)	0 (0.0)	0 (0.0)	35 (17.1)
Disagree	2 (50.0)	12 (15.0)	3 (12.0)	17 (20.2)	0 (0.0)	0 (0.0)	4 (33.3)	38 (18.5)
Strongly disagree	1 (25.0)	7 (8.8)	3 (12.0)	7 (8.3)	0 (0.0)	0 (0.0)	4 (33.3)	22 (10.7)
Had to reduce or give up exercise, *n* (%)	*n* = 4	*n* = 80	*n* = 25	*n* = 84	*n* = 0	*n* = 0	*n* = 12	*n* = 205
Strongly agree	0 (0.0)	25 (31.3)	6 (24.0)	21 (25.0)	0 (0.0)	0 (0.0)	1 (8.3)	53 (25.9)
Agree	2 (50.0)	31 (38.8)	4 (16.0)	28 (33.3)	0 (0.0)	0 (0.0)	3 (25.0)	68 (33.2)
Neither agree nor disagree	1 (25.0)	9 (11.3)	8 (32.0)	10 (11.9)	0 (0.0)	0 (0.0)	1 (8.3)	29 (14.1)
Disagree	1 (25.0)	11 (13.8)	5 (20.0)	16 (19.0)	0 (0.0)	0 (0.0)	3 (25.0)	36 (17.6)
Strongly disagree	0 (0.0)	4 (5.0)	2 (8.0)	9 (10.7)	0 (0.0)	0 (0.0)	4 (33.3)	19 (9.3)
Miss out on opportunities, *n* (%)	*n* = 4	*n* = 80	*n* = 25	*n* = 84	*n* = 0	*n* = 0	*n* = 12	*n* = 205
Strongly agree	0 (0.0)	17 (21.3)	5 (20.0)	15 (17.9)	0 (0.0)	0 (0.0)	0 (0.0)	37 (18.0)
Agree	1 (25.0)	31 (38.8)	8 (32.0)	33 (39.3)	0 (0.0)	0 (0.0)	4 (33.3)	77 (37.6)
Neither agree nor disagree	0 (0.0)	15 (18.8)	5 (20.0)	13 (15.5)	0 (0.0)	0 (0.0)	0 (0.0)	33 (16.1)
Disagree	3 (75.0)	12 (15.0)	5 (20.0)	14 (16.7)	0 (0.0)	0 (0.0)	3 (25.0)	37 (18.0)
Strongly disagree	0 (0.0)	5 (6.3)	2 (8.0)	9 (10.7)	0 (0.0)	0 (0.0)	5 (41.7)	21 (10.2)
Feel frustrated by the influence on lifestyle, *n* (%)	*n* = 4	*n* = 80	*n* = 25	*n* = 84	*n* = 0	*n* = 0	*n* = 12	*n* = 205
Strongly agree	0 (0.0)	15 (18.8)	6 (24.0)	13 (15.5)	0 (0.0)	0 (0.0)	1 (8.3)	35 (17.1)
Agree	0 (0.0)	25 (31.3)	7 (28.0)	36 (42.9)	0 (0.0)	0 (0.0)	2 (16.7)	70 (34.1)
Neither agree nor disagree	1 (25.0)	13 (16.3)	7 (28.0)	11 (13.1)	0 (0.0)	0 (0.0)	2 (16.7)	34 (16.6)
Disagree	3 (75.0)	21 (26.3)	3 (12.0)	14 (16.7)	0 (0.0)	0 (0.0)	2 (16.7)	43 (21.0)
Strongly disagree	0 (0.0)	6 (7.5)	2 (8.0)	10 (11.9)	0 (0.0)	0 (0.0)	5 (41.7)	23 (11.2)

Abbreviations: ABR, annualized bleeding rate; EQ-5D, EuroQol-5 Dimension; IQR, interquartile range; SD, standard deviation.

 © 2024. The Author(s).

Supplementary Table S2 CHESS PAEDs outcomes evaluated by country of origin

	Country					
	France	Germany	Italy	Spain	UK	Total
	n = 148	*n* = 112	*n* = 167	*n* = 168	*n* = 108	*N* = 703
ABR,						
n	147	112	166	161	105	691
Mean (SD)	1.56 (1.38)	1.64 (1.54)	2.91 (3.51)	2.81 (3.14)	10.99 (20.59)	3.62 (8.94)
Median (IQR)	1 (2)	1 (3)	2 (4)	2 (3)	4 (7)	2 (4)
ABR category, *n* (%)						
0	42 (28.4)	35 (31.3)	52 (31.1)	35 (20.8)	19 (17.6)	183 (26.0)
1	33 (22.3)	26 (23.2)	30 (18.0)	39 (23.2)	6 (5.6)	134 (19.1)
2+	73 (49.3)	51 (45.5)	85 (50.9)	94 (56.0)	83 (76.9)	386 (54.9)
ABR physician reported						
n	147	112	166	161	105	691
Mean (SD)	2.73 (3.96)	1.96 (1.42)	2.74 (3.46)	2.93 (2.85)	12.41 (22.72)	4.12 (9.93)
Median (IQR)	2 (2)	2 (2)	2 (3)	2 (3)	2 (18)	2 (3)
ABR category physician reported, *n* (%)						
0	14 (9.5)	14 (17.5)	37 (22.2)	17 (10.1)	13 (12.0)	95 (13.5)
	31 (20.9)	35 (31.3)	37 (22.2)	39 (23.2)	14 (13.0)	156 (22.2)
2+	103 (69.6)	63 (56.3)	93 (55.7)	112 (66.7)	81 (75.0)	452 (64.3)
% with zero bleeds (from physician-reported ABR), *n* (%)	14 (9.5)	14 (12.5)	37 (22.2)	17 (10.1)	13 (12.0)	95 (13.5)
Number of target joints						
n	148	112	167	168	108	703
Mean (SD)	0.23 (0.54)	0.13 (0.41)	0.15 (0.39)	0.27 (0.61)	0.19 (0.46)	0.20 (0.50)
Median (IQR)	0 (0)	0 (0)	0 (0)	0 (0)	0 (0)	0 (0)
Target joint number category, *n* (%)						
None	121 (81.8)	100 (89.3)	144 (86.2)	132 (78.6)	91 (84.3)	588 (83.6)
1 target joint	21 (14.2)	9 (8.0)	21 (12.6)	30 (17.9)	14 (13.0)	95 (13.5)
2+ target joints	6 (4.1)	3 (2.7)	2 (1.2)	6 (3.6)	3 (2.8)	20 (2.8)
Level of chronic pain, *n* (%)	*n* = 147	*n* = 112	*n* = 166	*n* = 161	*n* = 105	*n* = 691
No pain	53 (36.1)	59 (52.7)	58 (34.9)	68 (42.2)	47 (44.8)	285 (41.2)
Mild pain	68 (46.3)	44 (39.3)	80 (48.2)	67 (41.6)	37 (35.2)	296 (42.8)
Moderate pain	26 (17.7)	9 (8.0)	28 (16.9)	25 (15.5)	20 (19.0)	108 (15.6)
Severe pain	0 (0.0)	0 (0.0)	0 (0.0)	1 (0.6)	1 (1.0)	2 (0.3)
Anxiety, *n* (%)	19 (12.9)	2 (1.8)	11 (6.6)	17 (10.6)	7 (6.7)	56 (8.1)
Depression, *n* (%)	0 (0.0)	1 (0.9)	2 (1.2)	8 (5.0)	2 (1.9)	13 (1.9)
EQ-5D-Y score for children >8,						
n	21	32	13	19	36	121
Mean (SD)	0.73 (0.15)	0.65 (0.26)	0.54 (0.37)	0.60 (0.36)	0.68 (0.29)	0.65 (0.29)
Median (IQR)	0.73 (0.11)	0.69 (0.2)	0.69 (0.21)	0.76 (0.54)	0.67 (0.47)	0.69 (0.22)
Had to reduce or give up social activities, *n* (%)	*n* = 33	*n* = 44	*n* = 26	*n* = 31	*n* = 39	*n* = 173
Strongly agree	8 (24.2)	4 (9.1)	0 (0.0)	0 (0.0)	0 (0.0)	12 (6.9)
Agree	8 (24.2)	8 (18.2)	6 (23.1)	10 (32.3)	1 (2.6)	33 (19.1)

 © 2024. The Author(s).

Supplementary Table S2 (*Continued*)

	Country					
	France	Germany	Italy	Spain	UK	Total
	n = 148	*n* = 112	*n* = 167	*n* = 168	*n* = 108	*N* = 703
Neither agree nor disagree	4 (12.1)	9 (20.5)	8 (30.8)	4 (12.9)	10 (25.6)	35 (20.2)
Disagree	12 (36.4)	19 (43.2)	11 (42.3)	13 (41.9)	26 (66.7)	81 (46.8)
Strongly disagree	1 (3.0)	4 (9.1)	1 (3.8)	4 (12.9)	2 (5.1)	12 (6.9)
Had to reduce or give up exercise, *n* (%)	*n* = 33	*n* = 44	*n* = 26	*n* = 32	*n* = 39	*n* = 174
Strongly agree	8 (24.2)	0 (0.0)	0 (0.0)	0 (0.0)	0 (0.0)	8 (4.6)
Agree	1 (3.0)	5 (11.4)	6 (23.1)	5 (15.6)	5 (12.8)	22 (12.6)
Neither agree nor disagree	3 (9.1)	12 (27.3)	10 (38.5)	9 (28.1)	14 (35.9)	48 (27.6)
Disagree	16 (48.5)	23 (52.3)	9 (34.6)	13 (40.6)	13 (33.3)	74 (42.5)
Strongly disagree	5 (15.2)	4 (9.1)	1 (3.8)	5 (15.6)	7 (17.9)	22 (12.6)
Miss out on opportunities, *n* (%)	*n* = 33	*n* = 44	*n* = 26	*n* = 32	*n* = 39	*n* = 174
Strongly agree	6 (18.2)	0 (0.0)	0 (0.0)	0 (0.0)	1 (2.6)	7 (4.0)
Agree	3 (9.1)	8 (18.2)	4 (15.4)	5 (15.6)	6 (15.4)	26 (14.9)
Neither agree nor disagree	5 (15.2)	17 (38.6)	11 (42.3)	13 (40.6)	13 (33.3)	59 (33.9)
Disagree	19 (57.6)	16 (36.4)	11 (42.3)	11 (34.4)	16 (41.0)	73 (42.0)
Strongly disagree	0 (0.0)	3 (6.8)	0 (0.0)	3 (9.4)	3 (7.7)	9 (5.2)
Feel frustrated by the influence on lifestyle, *n* (%)	*n* = 33	*n* = 44	*n* = 26	*n* = 32	*n* = 39	*n* = 174
Strongly agree	3 (9.1)	0 (0.0)	0 (0.0)	1 (3.1)	0 (0.0)	4 (2.3)
Agree	5 (15.2)	6 (13.6)	2 (7.7)	10 (31.3)	2 (5.1)	25 (14.4)
Neither agree nor disagree	7 (21.2)	22 (50.0)	9 (34.6)	7 (21.9)	15 (38.5)	60 (34.5)
Disagree	17 (51.5)	15 (34.1)	13 (50.0)	11 (34.4)	18 (46.2)	74 (42.5)
Strongly disagree	1 (3.0)	1 (2.3)	2 (7.7)	3 (9.4)	4 (10.3)	11 (6.3)
Parent/guardian providing care for a pediatric relative with hemophilia, *n* (%)	*n* = 29	*n* = 43	*n* = 26	*n* = 34	*n* = 39	*n* = 171
Yes	26 (89.7)	13 (30.2)	24 (92.3)	26 (76.5)	31 (79.5)	120 (70.2)
No	3 (10.3)	30 (69.8)	2 (7.7)	8 (23.5)	8 (20.5)	51 (29.8)
Hours spent caring in a week						
N	26	13	22	26	31	118
Mean (SD)	9.24 (11.81)	18.92 (11.49)	18.36 (10.95)	49.58 (37.14)	11.68 (7.52)	21.54 (24.72)
Median (IQR)	4 (10)	20 (9)	15 (20)	59 (70)	9 (14)	12 (18)
Do your duties prevent you from working / working more hours? *n* (%)	*n* = 19	*n* = 13	*n* = 21	*n* = 17	*n* = 23	*n* = 93
Yes	0 (0.0)	1 (7.7)	2 (9.5)	5 (29.4)	12 (52.2)	20 (21.5)
No	19 (100.0)	10 (76.9)	15 (71.4)	7 (41.2)	9 (39.1)	60 (64.5)
Don't know	0 (0.0)	2 (15.4)	4 (19.0)	5 (29.4)	2 (8.7)	13 (14.0)

Abbreviations: ABR, annualized bleeding rate; EQ-5D, EuroQol-5 Dimension; IQR, interquartile range; SD, standard deviation.

 © 2024. The Author(s).
